# Spectroscopic Properties of Tb^3+^ Ions in TbF_3_-Doped CaF_2_ Crystals

**DOI:** 10.3390/ma19040801

**Published:** 2026-02-18

**Authors:** Irinuca Bodea, Marius Stef, Carla Schornig, Gabriel Buse, Philippe Veber, Daniel Vizman

**Affiliations:** 1 Department of Physics, West University of Timisoara, Bd. Vasile Parvan 4, 200223 Timisoara, Romania; irinuca.bodea99@e-uvt.ro (I.B.); carla.schornig@e-uvt.ro (C.S.); philippe.veber@e-uvt.ro (P.V.); daniel.vizman@e-uvt.ro (D.V.); 2 Institute for Advanced Environmental Research, West University of Timisoara, 300086 Timisoara, Romania; gabriel.buse@e-uvt.ro

**Keywords:** fluoride crystals, thulium, optical spectroscopy, Judd–Ofelt analysis, luminescence

## Abstract

**Highlights:**

**What are the main findings?**

- Tb^3+^-doped CaF_2_ crystals exhibit intense and stable green emission dominated by the ^5^D_4_ → ^7^F_5_ transition.
- Judd–Ofelt analysis provides radiative parameters of Tb_3+_-doped CaF_2_ crystal.
- The measured quantum efficiency increases from 31.4% (1 mol%) to 71.2% (5 mol%) and 73.9% (10 mol%).

**What are the implications of the main findings?**

- Efficient green emission is achieved at moderate–high Tb^3+^ concentrations without strong quenching.
- Cross-relaxation (^5^D_3_ → ^5^D_4_) enables effective population of the ^5^D_4_ emitting level.
- CaF_2_:Tb^3+^ combines millisecond lifetimes (~4.4–6.7 ms) with high QE, suitable for photonic applications.

**Abstract:**

Tb^3+^-doped CaF_2_ single crystals are attractive materials for green photonic applications due to their low phonon energy, high optical transparency, and efficient Tb^3+^ emission. In this work, CaF_2_ single crystals doped with different TbF_3_ concentrations (1, 5, and 10 mol%) were grown and systematically investigated in order to clarify the concentration-dependent spectroscopic behavior of Tb^3+^ ions in a fluorite host. Optical absorption spectroscopy, Judd–Ofelt analysis, steady-state and time-resolved photoluminescence, colorimetric evaluation, and emission cross-section and gain calculations were employed. Judd–Ofelt intensity parameters typical of fluoride hosts were obtained, enabling the calculation of radiative transition probabilities and lifetimes. The emission spectra are dominated by intense green luminescence from the ^5^D_4_ → ^7^F_5_ transition, while the absence of ^5^D_3_ emission is attributed to efficient cross-relaxation processes. Fluorescence lifetimes in the millisecond range show slight changes with Tb^3+^ concentration. Quantum efficiency increases from low to intermediate concentrations and tends to saturate at higher doping levels. CIE 1931 chromaticity coordinates confirm stable green emission, while emission cross-sections and gain parameters reveal a highest value for orange emission of 10 mol% TbF_3_-doped CaF_2_ crystal. These results indicate that CaF_2_:Tb^3+^ single crystals are promising materials for photonic applications.

## 1. Introduction

Rare-earth-doped materials are key enabling materials for a wide range of photonic applications, including solid-state lasers [1,2,3,4,5,6], wavelength converters [7], magneto-optical components [8,9], scintillators [10,11,12], and luminescent phosphors [13,14,15,16,17,18,19]. In particular, fluorite-type hosts such as CaF_2_ [20,21,22,23,24,25], BaF_2_ [26,27,28,29] or KY_3_F_10_ [30] have attracted sustained interest for visible and near-infrared photonics, due to their low multiphonon relaxation rates and high optical quality, which enable efficient emission and amplification processes, or magneto-optical properties [8]. Tb^3+^-activated fluoride materials are especially relevant for green photonics, optical signaling, and display-related technologies, due to the intense ^5^D_4_ → ^7^F_J_ (J = 3, 4, 5) emission manifold and the possibility of tailoring radiative properties through dopant concentration control [9,20,31,32,33,34,35,36,37].

From a spectroscopic point of view, the performance of rare-earth-activated photonic materials is dictated by the electronic structure of the dopant ions and by their interaction with the host lattice. The optical transitions of Tb^3+^ ions arise from the 4*f*^8^ electronic configuration and are characterized by a rich manifold of narrow 4*f*−4*f* transitions, whose energies and relative intensities have been thoroughly established through fundamental spectroscopic investigations and tabulations of energy levels and radiative parameters [38,39,40]. In particular, the ^5^D_4_ excited state of Tb^3+^ is responsible for intense green emission through the ^5^D_4_ → ^7^F_J_ (J = 6−3) transitions, while the higher-lying ^5^D_3_ state can contribute blue emission under suitable excitation and concentration regimes, making Tb^3+^ a versatile activator for visible photonics [41,42,43,44].

Compared to LnF_3_ fluorides, CaF_2_ offers a cubic crystal structure with higher symmetry, optical isotropy, negligible birefringence, and a broader transparency window, together with superior crystal growth stability and lower scattering losses, making it an excellent host for Tb^3+^ ions [40].

When embedded in crystalline hosts, the spectroscopic properties of Tb^3+^ are modulated by crystal-field splitting, site symmetry, and phonon-assisted relaxation processes, which together govern emission bandwidths, radiative lifetimes, and quantum efficiencies. Fluoride lattices are particularly advantageous in this respect, as their low maximum phonon energies significantly suppress multiphonon non-radiative decay, thereby preserving high radiative efficiencies of Tb^3+^ emission compared to oxide hosts [45,46,47]. Consequently, fluoride-based systems have been investigated as efficient hosts for Tb^3+^-activated luminescent materials, lasers, and magneto-optical components, with various studies demonstrating the sensitivity of Tb^3+^ emission characteristics to the local structural environment and dopant concentration. However, the incorporation of trivalent rare-earth ions into the divalent Ca^2+^ sublattice is intrinsically accompanied by charge-compensation mechanisms, most commonly involving fluorine interstitials or complex defect clusters, which profoundly influence the local environment of the optically active centers [22,23,29,48]. As a result, even at relatively low dopant concentrations, CaF_2_:RE^3+^ systems may exhibit multiple crystallographic sites and defect-associated centers, leading to inhomogeneous broadening of optical bands and non-trivial variations in luminescence dynamics [3,22,31]. These defect-related effects become particularly important for Tb^3+^-doped CaF_2_, where the balance between isolated ions and clustered centers determines the efficiency and spectral distribution of the ^5^D_4_ and ^5^D_3_ emissions. Experimental studies have shown that increasing the Tb^3+^ concentration can promote ion–ion interactions, cross-relaxation processes, and energy migration toward quenching centers, thereby modifying radiative lifetimes and emission intensities [9,17,20,21,30,36]. Moreover, the spatial distribution of RE^+^ ions—affected by growth conditions, segregation phenomena, and post-growth treatments—has been identified as a key factor governing the reproducibility and performance of photonic materials [49]. These considerations underline the necessity of a detailed spectroscopic analysis that explicitly accounts for defect chemistry and concentration-dependent effects when assessing the optical properties of Tb^3+^ in CaF_2_.

A quantitative assessment of the optical performance of Tb^3+^-doped CaF_2_ requires reliable determination of radiative transition probabilities, branching ratios, and intrinsic lifetimes, parameters that are essential for evaluating the suitability of these materials for photonic applications. In this context, the Judd–Ofelt (J–O) formalism has been widely adopted as a standard theoretical framework to extract radiative properties of rare-earth ions in solids from experimental absorption and emission data [50,51,52,53,54]. When applied to fluoride hosts, the J–O analysis benefits from reduced multiphonon contributions and relatively narrow spectral features, allowing a more direct correlation between intensity parameters and the local symmetry and covalency of the rare-earth sites [3,13,22,24,29].

Nevertheless, the application of the J–O approach to Tb^3+^-doped CaF_2_ is not straightforward and remains subject to several methodological challenges. The coexistence of multiple Tb^3+^-related centers, arising from different charge-compensation schemes and defect complexes, can lead to ambiguities in the assignment of absorption bands and to uncertainties in the extracted intensity parameters [22,26]. Furthermore, recent studies have highlighted that inaccuracies in absorption cross-section determination, spectral overlap between transitions, and the neglect of site multiplicity may introduce significant systematic errors in J–O-derived radiative parameters, particularly in fluorite-type lattices [3,29]. Despite the extensive body of literature dedicated to rare-earth-doped fluorides, the spectroscopic characterization of Tb^3+^ in CaF_2_ remains fragmented, with most studies addressing either specific emission features, particular concentration regimes, or selected material forms such as thin films [37,42] and nanostructures [33]. Moreover, recent advances in spectroscopic methodologies and data analysis, including refined absorption cross-section measurements and critical assessments of Judd–Ofelt uncertainties, have highlighted the need for revisiting classical Tb^3+^:CaF_2_ systems using updated experimental protocols and interpretative frameworks 53,54]. This is especially relevant in view of the renewed interest in CaF_2_-based photonic materials for visible emission, laser-related applications, and radiation-resistant optical components, where accurate and reproducible radiative parameters are essential for material optimization and device design.

In this context, the present work aims to provide a systematic spectroscopic analysis of Tb^3+^ ions in TbF_3_-doped CaF_2_ single crystals. By combining steady-state absorption and photoluminescence spectroscopy with time-resolved measurements and a careful radiative analysis, this study seeks to quantify luminescence parameters relevant to photonic applications in the visible spectral region and to investigate the influence of dopant concentration on the optical response of Tb^3+^:CaF_2_. The results are intended to contribute to a more coherent understanding of Tb^3+^-activated fluorite materials and to provide a reliable reference for future developments in fluoride-based photonics.

## 2. Materials and Methods

**Figure 1:**
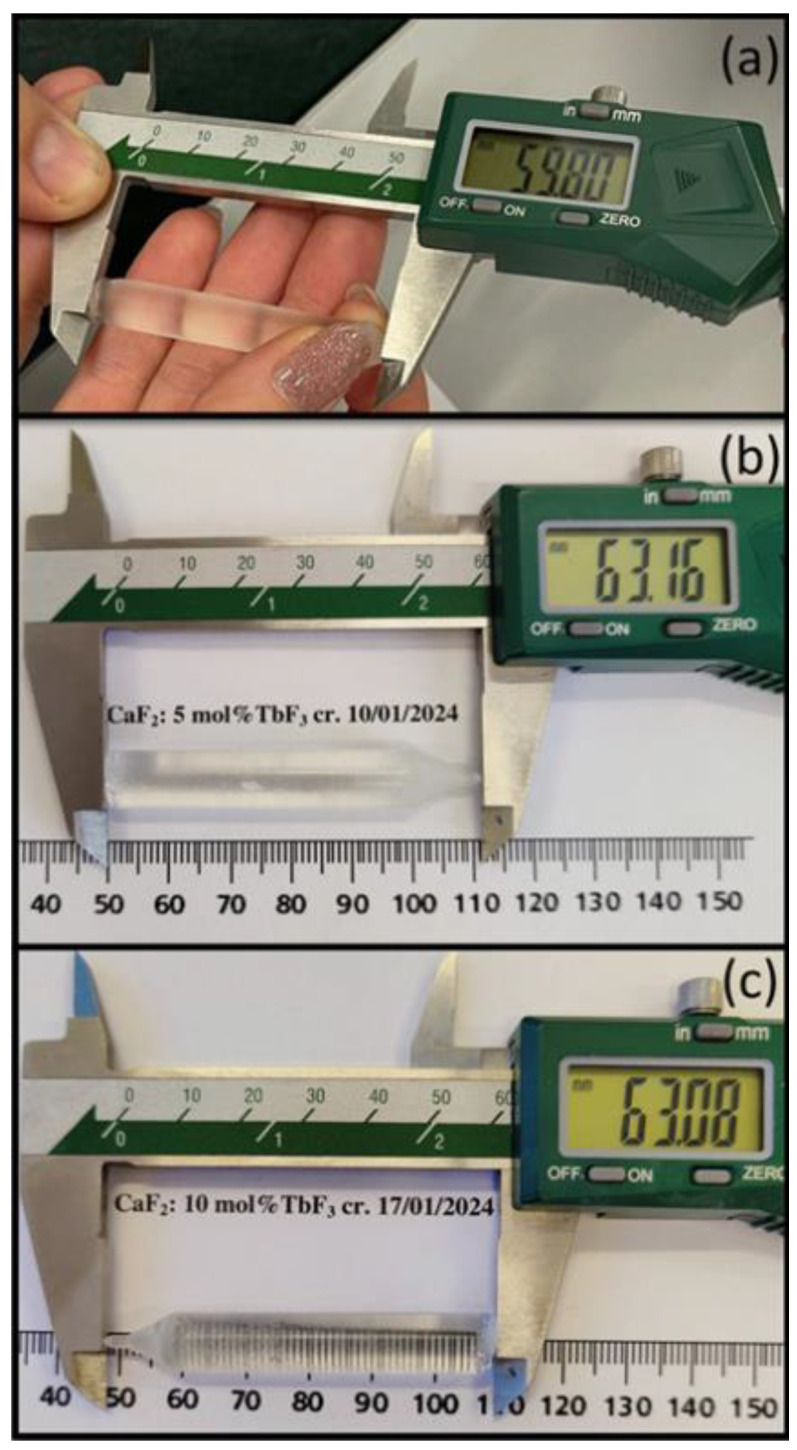
Photographs of CaF_2_ single crystals doped with: (**a**) 1 mol% TbF_3_; (**b**) 5 mol% TbF_3_; (**c**) 10 mol% TbF_3_. The measured crystal lengths are indicated.

Single crystals of terbium-doped calcium fluoride (CaF_2_:Tb^3+^) were grown using vertical Bridgman–Stockbarger method under high vacuum conditions (10^−1^ Pa) in a pure graphite crucible, following procedures optimized for rare-earth doped CaF_2_ crystals [3,24,49]. High-purity CaF_2_ and TbF_3_ powders (optical grade) from Merck (99.99%) were used as the raw material. The nominal terbium concentrations investigated in the present study were 1, 5 and 10 mol% TbF_3_ added to the melt. In the CaF_2_ fluorite lattice, RE^3+^ ions predominantly substitute Ca^2+^ sites, leading to an extra positive charge. Charge compensation is mainly achieved through the formation of interstitial fluoride ions (F^−^ᵢ), resulting in defect complexes of the type Tb^3+^–F^−^ᵢ [22,26]. At higher dopant concentrations, such charge-compensation mechanisms may induce multisite and local structural distortions, while preserving the overall fluorite crystal structure. By using laser-induced breakdown spectroscopy (LIBS), the Tb^3+^ concentration in the crystals is determined to be 2.437, 11.9 and 22.14 × 10^20^ ions/cm^3^ corresponding to the nominal concentration of 1, 5 and 10 mol% TbF_3._ Crystal growth was performed at temperatures exceeding the melting point of CaF_2_ (1381 °C), followed by controlled cooling to promote single-crystal formation and reduce thermal stress. Particular attention was paid to the solidification rate (~3 °C/min) and thermal gradient (~12 °C/cm), as these parameters are known to strongly influence defect formation and dopant distribution in fluorite-type crystals [49,50]. Figure 1 illustrates representative samples corresponding to different terbium concentrations. The crystals exhibit good transparency and no visible cracks, confirming the suitability of the growth conditions for high-quality fluoride crystals. As shown in Figure 1a–c, the absence of visible cracks or strong light scattering indicates a good overall optical quality, which is a prerequisite for reliable spectroscopic investigations.

After growth, the as-grown crystals were cleaved along the (111) crystallographic directions into optically flat samples with ~2 mm thickness suitable for spectroscopic investigations.

In order to investigate the etch pit morphology and evaluate the dislocation density in Tb^3+^-doped CaF_2_ single crystals, a chemical etching technique was employed. Freshly cleaved samples with surfaces oriented along the (111) crystallographic plane were immersed in a 4N hydrochloric acid (HCl) solution for 5 min at 60 °C. The etching process was carried out in a temperature-controlled water bath to ensure stable and reproducible conditions. After etching, the samples were thoroughly rinsed with distilled water, immediately dried using filter paper, and subsequently examined by optical microscopy. The etched surfaces were analyzed using an optical microscope equipped with an Olympus UC-30 image acquisition and processing module. This system enabled detailed visualization of etch pits, as well as quantitative measurements of their dimensions and the determination of the dislocation density in the Tb^3+^-doped CaF_2_ crystals.

**Figure 2:**
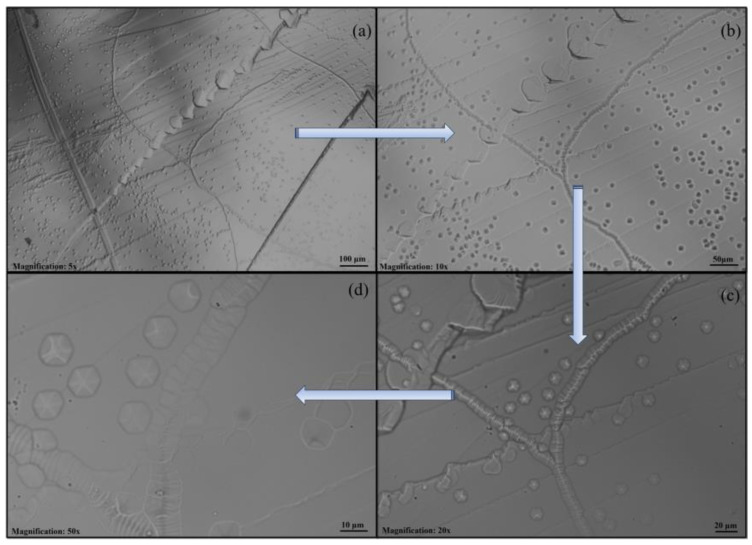
Dislocations in CaF_2_:1 mol% TbF_3_ crystal observed on the (111) fresh cleaved plane under various optical magnification: (**a**) 5×, (**b**) 10×, (**c**) 20× and (**d**) 40×.

Figure 2 shows the dislocations in CaF_2_:1 mol% TbF_3_ single crystals observed on the averaged (111) cleavage plane under various magnifications from 5 × to 40 ×. CaF_2_:Tb^3+^ single crystals doped with 1, 5, and 10 mol% TbF_3_ exhibited dislocation densities of 6.3 × 10^3^, 2.5 × 10^3^, and 17 × 10^3^ dislocations/cm^2^, respectively, these relatively low values confirming the high crystalline quality of the samples. The etch pit shape observed on the cleavage planes in the vicinity of (111) directions is slightly distorted hexagons, exhibiting thus a 3-fold axis in accordance to the (111) direction of cubic CaF_2_. They have a quasi-uniform spatial distribution (Figure 2a–d). A similar etch pit shape was previously reported for BaF_2_:Tm^3+^ crystals, where the dislocation density reached 3.9 × 10^3^ dislocations/cm^2^ [29].

The room-temperature optical absorption spectra, in the UV–VIS domain, were recorded using a Shimadzu 1650 PC from Shimadzu, Kyoto, Japan, spectrophotometer. The IR absorption spectrum was obtained with a Nexus 470 FTIR spectrophotometer. Room-temperature visible luminescence spectra and the time-resolved measurements were recorded by FLS 1000–Edinburgh Instruments spectrofluorometer using Xe lamp as the excitation source and a scan slit of 1 nm.

Such dislocation structures are commonly reported in CaF_2_ crystals [55] and are generally associated with lattice strain induced by aliovalent substitution of Ca^2+^ by Tb^3+^ ions and the corresponding charge-compensation mechanisms. Although these defects may locally disrupt the crystal field environment of Tb^3+^ ions, their overall density in the present samples remains sufficiently low to allow reliable optical and spectroscopic characterization. The combined macroscopic transparency and controlled defect structure confirm that the grown crystals are appropriate for detailed spectroscopic analysis of Tb^3+^ luminescence properties.

Optical absorption spectra were recorded at room temperature using a Shimadzu 1650PC spectrophotometer on cleaved crystal samples with parallel faces in order to minimize scattering losses and interference effects. The absorption measurements were performed in the 190–1100 nm spectral range relevant for Tb^3+^ excitation, enabling identification of the characteristic 4*f*–4*f* transitions originating from the ^7^F_6_ ground state. The near-infrared absorption spectra were registered using a Nexus 470 FTIR spectrophotometer.

Photoluminescence (PL) spectra were measured using FLS1000-Edinburgh Instruments spectrofluorometer under selective excitation into the Tb^3+^ absorption bands, using Xe as the excitation source. The emitted light was collected in a right-angle geometry and analyzed with a calibrated detection system to ensure reliable comparison of relative emission intensities. Special attention was paid to the ^5^D_4_ → ^7^F_J_ emission manifold, which is of primary interest for visible photonic applications.

Time-resolved luminescence measurements were carried out to determine the decay dynamics of the excited Tb^3+^ states. Luminescence decay curves were acquired following pulsed excitation provided by a Xe flash lamp and fitted using standard exponential models, providing experimental lifetimes that were subsequently used for radiative and non-radiative analysis. The quantum efficiency measurements were carried out using QYPro integrating a sphere module.

The Judd–Ofelt (JO) analysis was applied to the room-temperature absorption spectra of Tb^3+^-doped CaF_2_ crystals to extract the intensity parameters, which were subsequently used to calculate radiative transition probabilities, branching ratios, and radiative lifetimes [51,52].

## 3. Results

### 3.1. Optical Absorption Spectra

The room-temperature optical absorption spectra of Tb^3+^-doped CaF_2_ single crystals were recorded in the 240–6000 nm spectral range in order to identify the characteristic 4*f*–4*f* transitions of Tb^3+^ ions and to provide the basis for subsequent radiative analysis. The absorption spectra exhibit a series of narrow bands superimposed on a weakly varying background, which is typical for parity-forbidden intra-configurational transitions of trivalent rare-earth ions embedded in wide-bandgap fluoride hosts [9,17,20,36].

**Figure 3:**
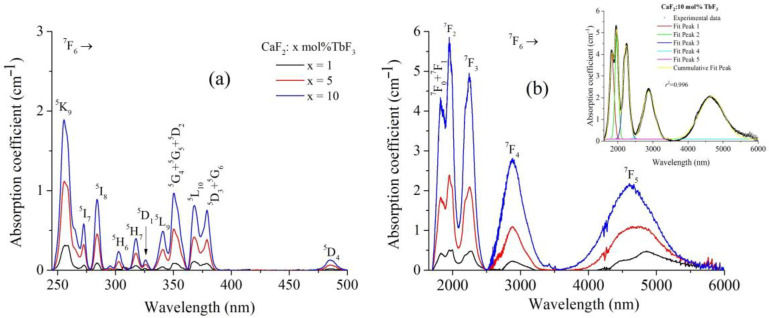
Room-temperature optical absorption spectra of CaF_2_:Tb^3+^ single crystals with nominal TbF_3_ concentrations of x = 1, 5, and 10 mol%. (**a**) UV–Vis spectra showing the characteristic 4*f*–4*f* transitions of Tb^3+^ ions from the ^7^F_6_ ground state. (**b**) NIR and mid-infrared absorption spectra with assigned ^7^F_J_ transitions. The inset shows a multi-peak Gaussian fitting of the infrared absorption spectrum for the CaF_2_:10 mol% TbF_3_ crystal.

Figure 3 shows the room-temperature optical absorption spectra of CaF_2_:Tb^3+^ single crystals with different nominal TbF_3_ concentrations (*x* = 1, 5, and 10 mol%), expressed as absorption coefficient as a function of wavelength. The spectra cover a wide spectral range and are presented in two panels: (a) the ultraviolet–visible region (≈240–500 nm) and (b) the near- and mid-infrared region (≈1800–6000 nm). In the UV–Vis range (Figure 3a), the absorption spectra are dominated by a series of narrow, well-resolved bands characteristic of intra-configurational 4*f*–4*f* transitions of Tb^3+^ ions originating from the ^7^F_6_ ground state. The observed absorption features can be assigned to transitions toward the ^5^K_J_, ^5^L_J_, ^5^G_J_, ^5^D_J_, ^5^H_J_ and ^5^I_J_ multiplets, as indicated in the figure. The spectral positions of these bands remain essentially unchanged with increasing Tb^3+^ concentration, indicating that the local crystal-field environment of Tb^3+^ ions is not significantly modified within the investigated doping range. On the other hand, the absorption intensity increases systematically with Tb^3+^ concentration, reflecting the higher density of optically active centers in the CaF_2_ lattice. The relatively narrow bands confirm the weak coupling between the 4*f* electrons and the host lattice phonons, which is typical for rare-earth ions in fluoride hosts [20]. The near-infrared absorption spectra (Figure 3b) show broader absorption bands compared to the UV–Vis region, with bands centered at approximately 1.8 μm, 2.0 μm, 2.2 μm, 2.8 μm, and a broad band around 4.6 μm. These bands are attributed to transitions from the ^7^F_6_ ground state to low-lying excited multiplets of Tb^3+^, labeled in the figure as ^7^F_J_ transitions. As in the UV–Vis region, the absorption intensity in the infrared increases monotonically with Tb^3+^ concentration, while no significant spectral shifts are observed, supporting a homogeneous incorporation of Tb^3+^ ions into the CaF_2_ host. The inset in Figure 3b presents a representative multi-peak fitting of the infrared absorption spectrum for the CaF_2_:10 mol% TbF_3_ sample. The experimental spectrum is accurately reproduced by a sum of individual Gaussian components, yielding a very good fit quality (correlation coefficient, *R*^2^ ≈ 0.996). This reliable deconvolution of overlapping absorption bands enables accurate determination of integrated absorption intensities, which are subsequently used as input parameters for the JO analysis and for the calculation of radiative transition probabilities and branching ratios. The band positions are in good agreement with the energy level scheme reported for Tb^3+^ ions in crystalline environments [17,20,36]. No additional broad absorption features attributable to impurity phases or secondary phases were detected, indicating a high optical quality of the investigated crystals.

### 3.2. Judd–Ofelt Analysis

The Judd–Ofelt (JO) theory is a well-established approach for the quantitative evaluation of 4*f*–4*f* transition intensities of rare-earth ions embedded in solid-state hosts [51,52]. Within this framework, the spectroscopic behavior of rare-earth ions is described by three phenomenological intensity parameters, Ω_2_, Ω_4_, and Ω_6_, which are determined by fitting experimental absorption data. Once obtained, the JO parameters allow the calculation of radiative properties, including electric dipole oscillator strengths, emission cross-sections, radiative transition probabilities, radiative lifetimes, and branching ratios. In the present study, the absorption spectra were used to determine the absorption cross-section, σ_abs_(λ), according to:

(1) $$\sigma_{abs}=\frac{O.D.ln(10)}{l\times N}$$

where l is the sample thickness, O.D. is the optical density, and *N* denotes the concentration of Tb^3+^ ions. In the JO formalism, the electric dipole line strength for a transition between an initial state |J〉 and a final state |J′〉 is expressed as: –

(2) $${\left(S_{JJ\prime }^{DE}\right)}_{calc}=\sum_{t=2,4,6}\Omega_{t}{\left|\left\langle 4f^{n}\alpha LSJ|\left|U^{t}\right||4f^{n}\alpha \prime L\prime S\prime J\prime \right\rangle \right|}^{2}$$

The magnetic dipole contribution to the line strength is given by:

(3) $${\left(S_{JJ\prime }^{DM}\right)}_{calc}={\left(\frac{\hbar }{2mc}\right)}^{2}{\left|\left\langle 4f^{n}\alpha LSJ|\left|L+2S\right||4f^{n}\alpha \prime L\prime S\prime J\prime \right\rangle \right|}^{2}$$

The reduced matrix elements,$\left|\left\langle 4f^{n}\alpha LSJ|\left|U^{t}\right||4f^{n}\alpha \prime L\prime S\prime J\prime \right\rangle \right|$ required for the evaluation of line strengths were taken from Kaminskii and tabulated in Table 1 [40]. In the present JO analysis, only electric dipole-allowed transitions were considered in the fitting procedure. The experimental line strengths, derived from absorption measurements, are related to the integrated absorption cross-sections by:

(4) $${\left(S_{JJ\prime }^{DE}\right)}_{meas}=S_{JJ\prime }^{meas}-\left(\frac{9n^{2}}{{\left(n^{2}+2\right)}^{2}}\right){\left(S_{JJ\prime }^{DM}\right)}_{calc}$$

where $S_{JJ\prime }^{meas}=\left(\frac{9n}{{\left(n^{2}+2\right)}^{2}}\right)\left(\frac{3hc\epsilon_{0}}{2\pi^{2}e^{2}}\right)\left(\frac{2J+1}{\lambda_{m}}\right)\int \sigma_{abs}(\lambda )d\lambda$.

where *n* is the refractive index of the host material determined by the Sellmeier’s equation [56]. The quality of the JO fit was evaluated by calculating the root-mean-square deviation, δ, between experimental and calculated line strengths:

(5) $$\delta =\sqrt{\frac{\sum_{i=1}^{q}{\left({\left(S_{JJ\prime }^{DE}\right)}_{calc}^{i}-{\left(S_{JJ\prime }^{DE}\right)}_{meas}^{i}\right)}^{2}}{q-p}}$$

where *q* = 3 and *p* = 4 are the number of absorption transitions strength selected, which correspond to the transitions: Transition, ^7^F_6_ → ^7^F_3_, ^7^F_2_, (^7^F_1_ + ^7^F_0_), ^5^D_4_. Using the optimized intensity parameters, the total radiative emission probability for a transition was obtained by summing the electric and magnetic dipole contributions:

(6) $$A_{JJ\prime }=A_{JJ\prime }^{ED}+A_{JJ\prime }^{MD}$$

from which the radiative lifetime of an excited level was calculated as:

(7) $$\tau_{rad}={\left(\sum_{JJ\prime }A_{JJ\prime }\right)}^{-1}$$

Finally, the branching ratio, defined as the relative probability of a radiative transition from a given excited level, was determined using:

(8) $$\beta_{JJ\prime }=\frac{A_{JJ\prime }}{\sum_{JJ\prime }A_{JJ\prime }}$$

Table 1 summarizes the squared reduced matrix elements, [U^(t)^]^2^ (t = 2, 4, 6), associated with the selected optical transitions of Tb^3+^ ions originating from the ^7^F_6_ ground state, together with the corresponding mean wavelengths.

Table 2 summarizes the experimental and calculated electric dipole line strengths for selected optical transitions of Tb^3+^ ions in CaF_2_ crystals with different nominal TbF_3_ concentrations (1, 5, and 10 mol%). The corresponding mean wavelengths and Tb^3+^ ion concentrations are also listed. The line strengths were derived from the absorption spectra and subsequently compared with the values obtained from the JO calculations. For all investigated concentrations, a reasonable agreement between the measured and calculated line strengths is observed. The root-mean-square deviation *δ*, reported for each concentration, decreases with increasing Tb^3+^ content, indicating an improvement in the overall quality of the JO fit at higher dopant concentrations. This behavior can be attributed to the enhanced signal-to-noise ratio of the absorption bands at higher terbium contents, which allows a more reliable determination of integrated absorption intensities.

Table 3 presents the JO intensity parameters Ω_2_, Ω_4_, and Ω_6_ obtained for CaF_2_:Tb^3+^ single crystals with different TbF_3_ concentrations (x = 1, 5, and 10 mol%), together with the spectroscopic quality factor, χ = Ω_4_/Ω_6_. For comparison, previously reported values for Tb^3+^-doped CaF_2_ and selected fluoride and oxide hosts are also included. The Ω_6_ parameter exhibits comparable values across the investigated concentration range, while Ω_2_ and Ω_4_ show more pronounced variations with Tb^3+^ content. The Ω_2_ parameter, which is often associated with local symmetry and covalency effects, remains relatively low compared to oxide hosts, consistent with the predominantly ionic character of the CaF_2_ lattice. In contrast, Ω_4_ displays a stronger dependence on dopant concentration, leading to variations in the Ω_4_/Ω_6_ ratio. The spectroscopic quality factor, χ, ranges from 0.52 to 1.46 for the CaF_2_:Tb^3+^ crystals investigated in this work, indicating moderate changes in the relative contributions of intermediate-rank electric dipole interactions with increasing Tb^3+^ concentration. These values are in good agreement with those reported by Liu et al. for 5% Tb doped in CaF_2_ crystals, around 1.18 [36]. When compared with literature data, the obtained JO parameters fall within the typical range reported for Tb^3+^-doped fluoride hosts and differ significantly from those of oxide and tungstate crystals, reflecting the lower phonon energies and reduced covalency of fluoride lattices [47].

Table 4 shows the radiative parameters associated with the ^5^D_4_ → ^7^F_J_ transitions of Tb^3+^ ions in CaF_2_ crystals doped with 1, 5, and 10 mol% TbF_3_. The table lists the transition wavelengths, radiative transition probabilities, *A_JJ′_*, branching ratios, *β_JJ_*_′_, and radiative lifetimes, τ_rad_, as derived from the JO analysis. For all investigated concentrations, the radiative decay from the ^5^D_4_ excited state is dominated by transitions to the ^7^F_5_ and ^7^F_3_ levels, which exhibit the highest radiative transition probabilities and branching ratios. In particular, the ^5^D_4_ → ^7^F_5_ transition shows the largest contribution to the total radiative decay, accounting for more than 50% of the total branching ratio, consistent with the intense green emission typically observed for Tb^3+^-activated materials [17,20,36,47]. The ^5^D_4_ → ^7^F_3_ transition represents the second most significant radiative channel, while transitions to the other ^7^F_J_ levels contribute more modestly.

The calculated radiative lifetimes of the ^5^D_4_ level decrease slightly with increasing Tb^3+^ concentration, reflecting the combined effect of increasing total radiative rates and concentration-dependent changes in the JO intensity parameters. The obtained lifetime values are in good agreement with previously reported data for Tb^3+^-doped fluoride crystals, as indicated by the literature references included in Table 4.

### 3.3. Emission Spectra

**Figure 4:**
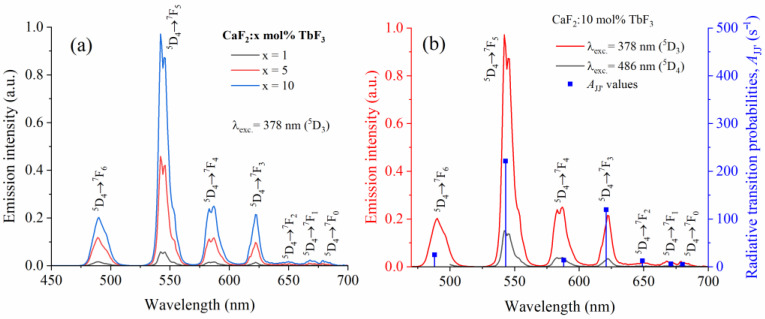
Room-temperature emission spectra of CaF^2^:Tb^3+^ single crystals. (**a**) Emission spectra of CaF_2_:*x* mol% TbF_3_ (*x* = 1, 5, and 10) under excitation at 378 nm (^5^D_3_ excitation), showing the characteristic ^5^D_4_ → ^7^F_J_ (J = 6–0) transitions of Tb^3+^ ions. (**b**) Emission spectrum of CaF_2_:10 mol% TbF_3_ measured under excitation at 378 nm (^5^D_3_) and 486 nm (^5^D_4_), together with the radiative transition probabilities, *A_JJ′_*, obtained from the JO analysis.

Figure 4 shows the room-temperature photoluminescence spectra of CaF_2_:Tb^3+^ single crystals with different TbF_3_ concentrations, recorded under selective excitation at 378 nm, corresponding to excitation into the ^5^D_3_ level. In Figure 4a, the emission spectra for *x* = 1, 5, and 10 mol% TbF_3_ are compared. All spectra are dominated by the characteristic green emission originating from the ^5^D_4_ → ^7^F_J_ (J = 6–0) transitions of Tb^3+^ ions. The most intense emission band is observed around 542 nm and is assigned to the ^5^D_4_ → ^7^F_5_ transition, which is known to be the dominant radiative channel in Tb^3+^-activated materials. Additional emission bands corresponding to the ^5^D_4_ → ^7^F_6−0_ transitions are clearly resolved. With increasing Tb^3+^ concentration, the overall emission intensity increases. The absence of detectable ^5^D_3_ → ^7^F_J_ emission bands, under excitation at 378 nm indicates an efficient non-radiative depopulation of the ^5^D_3_ level toward ^5^D_4_, consistent with well-known cross-relaxation processes in Tb^3+^-doped systems [36]. Figure 4b presents the emission spectrum of the CaF_2_:10 mol% TbF_3_ crystal under excitation at both 378 nm (^5^D_3_ excitation) and 486 nm (^5^D_4_ excitation), together with the radiative transition probabilities *A_JJ′_* derived from the JO analysis. The spectral shape remains essentially unchanged for the two excitation wavelengths, confirming that the emission originates predominantly from the ^5^D_4_ level, regardless of the excitation pathway. The relative intensities of the emission bands follow closely the calculated radiative transition probabilities, with the ^5^D_4_ → ^7^F_5_ transition exhibiting the highest *A_JJ_*_′_ value. This good agreement demonstrates the internal consistency between the experimental emission spectra and the *A_JJ_*_′_ values.

**Figure 5:**
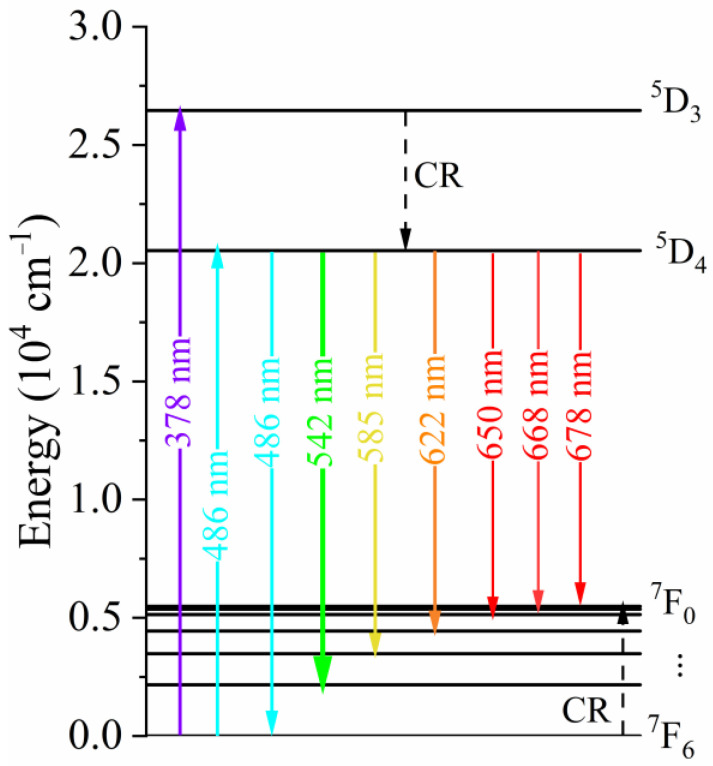
Simplified energy level diagram of Tb^3+^ ions in CaF_2_ showing the main excitation pathways (378 and 486 nm), the cross-relaxation (CR) process between the ^5^D_3_ and ^5^D_4_ levels, and the radiative ^5^D_4_ → ^7^F_J_ (J = 0–6) transitions responsible for the visible luminescence.

Figure 5 illustrates the simplified energy level diagram of Tb^3+^ ions in the CaF_2_ host, highlighting the main excitation, cross, relaxation (CR), and emission processes involved in the observed luminescence. The diagram includes the relevant excited states (^5^D_3_ and ^5^D_4_) and the lower-lying ^7^F_J_ manifolds, together with the corresponding transition energies expressed in cm^−1^. Excitation at 378 nm promotes Tb^3+^ ions to the ^5^D_3_ level, while excitation at 486 nm directly populates the ^5^D_4_ level. The diagram emphasizes the efficient non-radiative cross-relaxation (CR) process from the ^5^D_3_ to the ^5^D_4_ level, which leads to a rapid depopulation of the higher-lying ^5^D_3_ state. As a consequence, radiative emission originates from the ^5^D_4_ level, regardless of the excitation pathway. This CR luminescence mechanism has also been reported in Tb doped in CaF_2_ ceramics [20] and Gd_2_O_3_ glasses [12]. They conclude that the high Tb^3+^ concentrations make the distance of Tb^3+^–Tb^3+^ shorten and thus a fast CR from ^5^D_3_ to ^5^D_4_ energy level occurs. On the other hand, strong quenching of ^5^D_3_ → ^7^F_J_ emissions are observed. The downward arrows indicate the radiative transitions from the ^5^D_4_ excited state to the ^7^F_J_ (J = 0–6) ground-state multiplets, corresponding to the characteristic visible emission bands of Tb^3+^ ions. The strongest emission transitions are observed in the green spectral region, in agreement with the dominance of the ^5^D_4_ → ^7^F_5_ transition in the experimental emission spectra.

### 3.4. Fluorescence Dynamics and Internal Quantum Efficiency

**Figure 6:**
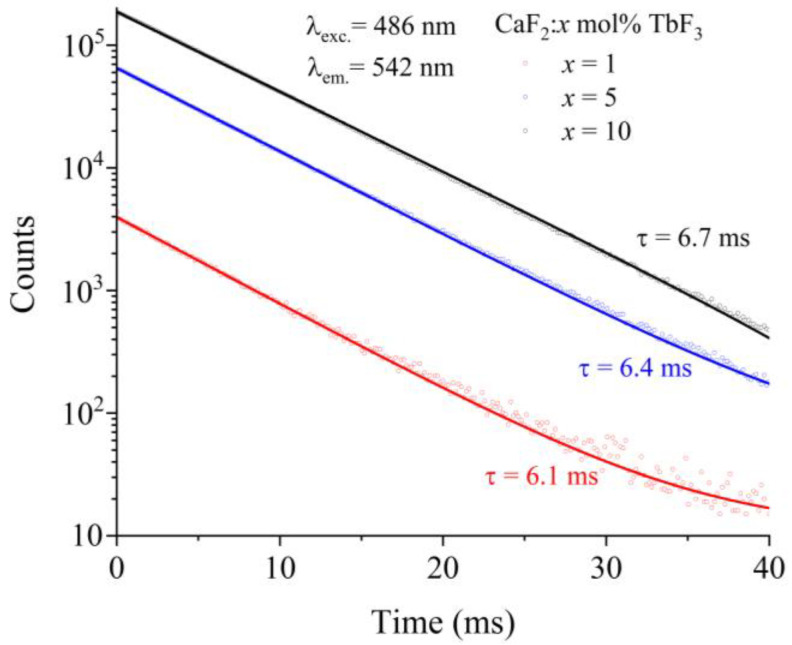
Decay curves of ^5^D_4_ level for various concentrations of Tb:CaF_2_ crystals at room temperature.

As seen in Figure 6, the photoluminescence decay curves of Tb:CaF_2_ crystal were obtained for the emissions measured under excitation at 486 nm (^5^D_4_ level). After being fitted, all the decay curves exhibit mono-exponential decaying behavior.

Table 5 summarizes the experimentally measured luminescence decay times, *τ*_meas_, of the ^5^D_4_ excited state of Tb^3+^ ions in CaF_2_ crystals doped with different TbF_3_ concentrations (1, 5, and 10 mol%). The decay times are reported for the main ^5^D_4_ → ^7^F_J_ emission transitions together with the corresponding emission wavelengths. The relative uncertainty associated with the fluorescence lifetime measurements reported in Table 5 ranges between 0.09% and 0.34%, indicating a high precision of the experimental lifetime measurements. For comparison, representative lifetime values reported in the literature for Tb^3+^-doped CaF_2_ and related fluoride systems are also included. The measured decay times lie predominantly in the range of approximately 4.4–6.5 ms, depending on the specific emission transition and terbium concentration. The ^5^D_4_ → ^7^F_5_ transition, which corresponds to the dominant green emission band, exhibits decay times of 6.1, 6.4, and 6.7 ms for 1, 5, and 10 mol% TbF_3_, respectively. The decay times are relatively similar for the different ^5^D_4_ → ^7^F_J_ transitions and different Tb concentrations. The comparison with literature data reveals that the lifetime values obtained in this work are generally consistent with those reported for other fluorite hosts, while some dispersion can be observed depending on crystal composition, morphology (bulk crystals versus nanostructures), and experimental conditions. Quantum efficiency values are also summarized in Table 5. These data offer a comprehensive understanding of the radiative efficiency and temporal behavior of each doped system, providing valuable parameters for optimizing phosphor performance in practical lighting applications. The internal quantum efficiency (IQE) is determined as a ratio between the number of photons emitted and number of photons absorbed. Number of photons emitted is proportional to the area under the emission spectrum. Number of photons absorbed is proportional to the difference between the area under the scattered band at the 486 nm excitation wavelength from the reference and that from the sample. The measured quantum efficiency of CaF_2_:Tb^3+^ crystals show a pronounced dependence on the TbF_3_ concentration. At 1 mol% TbF_3_, a relatively low quantum efficiency of 31.4% is obtained, indicating that non-radiative processes still play a significant role at low dopant concentrations, likely due to incomplete energy transfer and reduced probability of radiative recombination. A substantial increase in quantum efficiency is observed when the Tb_3_ concentration is raised to 5 mol%, where η reaches 71.2%. This improvement reflects a more efficient population of the ^5^D_4_ emitting level, consistent with the increased probability of energy migration toward radiative centers and with the effective suppression of competing non-radiative channels. At 10 mol% TbF_3_, the quantum efficiency further increases slightly to 73.9%, suggesting that the system approaches a saturation regime. Loiko et al. calculated the quantum efficiency of about 19% for Tb-doped KYb(WO_4_)_2_ crystal [47] and Wang et al. obtained 79.2% for Tb-doped CaYAlO_4_ crystal [45]. Furthermore, the quantum efficiency in LiTbF_4_ and TbCaF crystals were also calculated at about 65% and 21%, respectively [43], and 89.51% for Tb-doped CaF_2_ crystal [36]. This last value is higher than the one measured in this study of about 73.9%. Despite the higher dopant concentration, no significant concentration quenching is observed, indicating that the dominant cross-relaxation processes favor the population of the ^5^D_4_ level rather than introducing additional non-radiative losses. These results demonstrate that CaF_2_:Tb^3+^ crystals maintain high luminescence efficiency at elevated Tb^3+^ concentrations, which is particularly advantageous for green-emitting photonic applications.

### 3.5. CIE Color Coordinates

**Figure 7:**
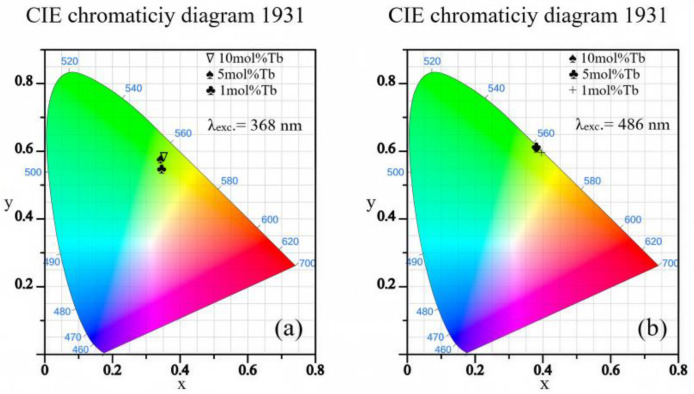
CIE 1931 chromaticity diagrams calculated from the room-temperature emission spectra of CaF_2_:Tb**^3+^** single crystals with nominal TbF_3_ concentrations of 1, 5, and 10 mol%. The emission points are located in the green region of the chromaticity space, indicating stable green luminescence dominated by the ^5^D**_4_** → ^7^F**_5_** transition of Tb**^3+^** ions under excitation at (**a**) 368 nm and (**b**) 486 nm.

The chromaticity characteristics of the Tb-doped CaF_2_ crystals were evaluated using the CIE 1931 color. The corresponding (x, y) coordinates were calculated using CIE 1931 web-based app [33]. These parameters provide information about the visual appearance and practical utility of the emitted light. The CIE color coordinates calculated for each sample are listed in Table 6. In addition, the positions of the emission colors for different dopant concentrations are graphically illustrated in Figure 7.

For all investigated samples, the emission points are located in the green region of the CIE 1931 diagram, reflecting the dominant contribution of the ^5^D_4_ → ^7^F_5_ transition of Tb^3+^ ions. A slight variation in the chromaticity coordinates with Tb^3+^ concentration is observed, indicating minor changes in the relative intensities of the individual ^5^D_4_ → ^7^F_J_ emission bands as the dopant content increases. However, the overall emission color remains stable and well confined within the green region of the chromaticity space. The comparison between the two panels highlights the reproducibility of the chromaticity coordinates under different excitation conditions, confirming that the emission color is predominantly governed by radiative transitions from the ^5^D_4_ level, independent of the excitation wavelength. These results demonstrate the good color stability of CaF_2_:Tb^3+^ single crystals and their suitability for green-emitting photonic applications. These results are in good agreement with those reported by Liu et al. [14], Kumari et al. [15], Wang et al. [45] and Loiko et al. [47].

### 3.6. Emission Cross-Section and Gain Parameter

To evaluate the emission cross-section corresponding to the measured luminescence bands, the Füchtbauer–Ladenburg equation [31] was used:

(9) $$\sigma_{em}\left(\lambda \right)\;=\;\frac{\lambda^{5}I(\lambda )}{8\pi {\left[n(\lambda )\right]}^{2}c\tau_{rad}\int \lambda I\left(\lambda \right)d\lambda }$$

where *I*(*λ*) is the emission intensity at each wavelength, τ_rad_ is the radiative lifetime of the upper level, *n* is the refractive index and *c* is the light speed.

**Figure 8:**
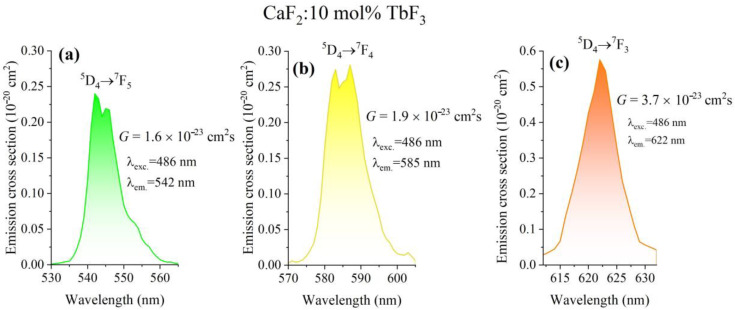
Emission cross-sections of CaF_2_:10 mol% TbF_3_: (**a**) the green emission around 542 nm, (**b**) the yellow emission at 585 nm and (**c**) the orange emission, 622 nm. On the figure are shown values obtained for gain parameter for each transition.

Figure 8 shows the emission cross-sections associated with the dominant ^5^D_4_ → ^7^F_J_ transitions of Tb^3+^ ions in CaF_2_ crystals (J = 5, 4, 3) doped with 10 mol% TbF_3_. The emission cross-sections were calculated from the experimental emission spectra using the Füchtbauer–Ladenburg approach and are shown for three representative transitions: (a) ^5^D_4_ → ^7^F_5_, (b) ^5^D_4_ → ^7^F_4_, and (c) ^5^D_4_ → ^7^F_3_. The emission bands exhibit well-defined spectral shapes centered at wavelengths characteristic of Tb^3+^ transitions. The two times higher emission cross-section is obtained for the orange emission of the ^5^D_4_ → ^7^F_3_ transition (Figure 8c). The emission cross-section data provide a quantitative measure of the radiative potential of the ^5^D_4_ → ^7^F_J_ transitions and confirm that CaF_2_:Tb^3+^ crystals show green-, yellow- and orange-emitting capabilities even at high Tb^3+^ concentrations (no concentration quenching is observed). To evaluate the laser performance, the optical gain parameter, *G* = σ_em_·τ_meas_, was calculated for the emissions of CaF_2_:TbF_3_ sample under excitation at 486 nm. The gain parameter provides a quantitative measure of the optical amplification potential of the investigated material. In the present study, the gain parameter was evaluated for the main ^5^D_4_ → ^7^F_J_ transitions (J = 5, 4, 3) of Tb^3+^ ions in CaF_2_ crystals doped with 10 mol% TbF_3_ (Figure 8). The highest gain value of 3.7×10^−23^ cm^2^s is obtained for the ^5^D_4_ → ^7^F_5_ transition (orange) of 10 mol% TbF_3_ content, in agreement with its dominant contribution to the green emission and with the JO-calculated radiative transition probabilities.

## 4. Discussions

This comprehensive spectroscopic investigation of Tb^3+^-doped CaF_2_ single crystals reveals a consistent picture in which the optical properties are governed by the interplay between the intrinsic electronic structure of Tb^3+^ ions, the fluorite host lattice, and concentration-dependent ion–ion interactions mediated by charge-compensation defects. By combining absorption spectroscopy, Judd–Ofelt analysis, emission measurements, fluorescence dynamics, colorimetric evaluation, emission cross-section and gain parameter calculations, a unified interpretation of the radiative behavior of Tb^3+^ in CaF_2_ can be established.

The optical absorption spectra presented in Section 3.1 exhibit the characteristic 4*f*–4*f* transitions of Tb^3+^ ions originating from the ^7^F_6_ ground state, with band positions that remain essentially unchanged across the investigated concentration range. This behavior indicates that the average crystal-field strength experienced by Tb^3+^ ions is not significantly modified by increasing TbF_3_ content, in agreement with previous reports on CaF_2_:RE^3+^ systems [16,20,22,32]. At the same time, the systematic increase in absorption intensity with concentration confirms the proportional incorporation of Tb^3+^ ions into the lattice. The absence of additional broad absorption features associated with secondary phases or strong defect-related bands suggests a high optical quality of the grown crystals. However, the observed variations in relative band intensities and linewidths are consistent with the presence of multiple Tb^3+^-related centers associated with different charge-compensation schemes, such as fluorine interstitials or defect clusters, which are known to occur in CaF_2_ doped with trivalent rare-earth ions [22,23,48]. These defect-related effects provide an important microscopic background for interpreting the subsequent radiative and non-radiative processes.

The Judd–Ofelt analysis (Section 3.2) yields intensity parameters Ω_2_, Ω_4_, and Ω_6_ that fall within the typical range reported for Tb^3+^-doped fluoride hosts and differ markedly from those of oxide or tungstate materials. In particular, the relatively low Ω_2_ values reflect the predominantly ionic character and high local symmetry of the CaF_2_ lattice, while the Ω_4_ and Ω_6_ parameters govern the strength of the ^5^D_4_ → ^7^F_J_ electric dipole transitions. The moderate variation in the Ω_4_/Ω_6_ ratio with Tb^3+^ concentration indicates subtle changes in the local environment and ion–ion interactions, rather than a fundamental alteration in the host lattice. The normalized emission spectra (Figure 4) confirm that the ratio of relative intensities remains relatively unchanged with the concentration of TbF_3_. Nevertheless, Ω_2_, being derived from absorption line strengths, remains highly sensitive to band overlap effects (especially due to the overlap of ^7^F_6_ → ^7^F_0,1,2,3_ absorption bands) and to possible inhomogeneities in dopant distribution along the crystals. The good agreement between experimental and calculated line strengths, together with decreasing rms deviations at higher concentrations, confirms the internal consistency of the JO formalism for describing Tb^3+^ absorption in CaF_2_. These results provide a reliable basis for the calculation of radiative transition probabilities, branching ratios, radiative lifetimes, emission cross section and gain parameter.

The emission spectra discussed in Section 3.3 are dominated by the ^5^D_4_ → ^7^F_J_ transitions, with the ^5^D_4_ → ^7^F_5_ green emission band representing the most intense radiative emission for all investigated concentrations. The absence of detectable ^5^D_3_ → ^7^F_J_ emission, even under direct excitation into the ^5^D_3_ level, indicates an efficient depopulation of ^5^D_3_ through cross-relaxation processes. This behavior is well explained by the cross-relaxation mechanism ^5^D_3_ + ^7^F_6_ → ^5^D_4_ + ^7^F_J_, which becomes increasingly efficient with increasing Tb^3+^ concentration, as previously reported for Tb^3+^-activated fluoride systems. In CaF_2_ crystal, the low phonon energy of the host lattice does not suppress this process, since cross-relaxation is governed by inter-ionic interactions rather than multiphonon relaxation. As a consequence, excitation energy is funneled efficiently toward the ^5^D_4_ level, leading to dominant green emission and enhanced radiative output. The close correspondence between the relative emission intensities and the JO-derived radiative transition probabilities further supports this interpretation and demonstrates the consistency between absorption-based modeling and emission measurements.

The fluorescence decay analysis (Section 3.4) reveals millisecond-range lifetimes characteristic of Tb^3+^ emission in low-phonon-energy fluoride hosts. The values obtained are close to each other and vary between 4.4 and 6.7 ms, indicating a slight dependence on Tb concentration.

The experimentally determined quantum efficiencies show a pronounced increase from low to intermediate Tb^3+^ concentrations, followed by a saturation tendency at higher doping levels. This behavior reflects an optimal balance between efficient population of the emitting ^5^D_4_ level and the onset of concentration-related losses, highlighting the robustness of CaF_2_ as a host for Tb^3+^-based green emitters. The CaF_2_ host is characterized by a low maximum phonon energy of approximately 456 cm^−1^, which effectively suppresses multiphonon non-radiative relaxation processes [57]. This low phonon energy is reflected in the long fluorescence lifetimes of the Tb^3+ 5^D_4_ level (~6 ms) observed in this work and plays a key role in maintaining high optical efficiency by favoring radiative decay channels.

The CIE 1931 chromaticity coordinates (Section 3.5) confirm that all investigated samples emit in the green region of the color space, with only minor variations as a function of Tb^3+^ concentration. This color stability is a direct consequence of the dominance of the ^5^D_4_ → ^7^F_5_ transition and the suppression of competing emission channels. Such stability is a desirable feature for applications in solid-state lighting, displays, and optical signaling.

The emission cross-sections derived in Section 3.6 further quantify the radiative potential of CaF_2_:Tb^3+^ crystals and allow direct comparison with other fluoride and oxide hosts [7,8,9,10,12,17,20,32,37]. The obtained values are consistent with the high radiative efficiencies observed experimentally of about 74% and underline the suitability of this system for visible photonic applications.

The obtained gain parameter values demonstrate that CaF_2_:Tb^3+^ crystals have a non-negligible optical amplification capability in the visible spectral range, particularly in the orange region associated with the ^5^D_4_ → ^7^F_3_ transition (two times higher than green or yellow emission). These results indicate that, beyond their luminescent performance, Tb^3+^-doped CaF_2_ crystals may be considered as potential candidates for visible photonic applications requiring moderate optical gain, provided that efficient population inversion can be achieved.

The present results demonstrate that CaF_2_:Tb^3+^ single crystals combine favorable spectroscopic properties, making them attractive candidates for photonic devices, magneto-optical components, and scintillation-related applications. The strong role of defect chemistry and cross-relaxation processes suggests that further optimization could be achieved through controlled growth conditions, segregation, co-doping strategies, or post-growth treatments aimed at tailoring the local environment of Tb^3+^ ions [7,36]. Future studies should focus on a more detailed correlation between defect structures and luminescence dynamics and the exploration of Tb^3+^-based co-doped systems to enhance emission efficiency and spectral tunability.

## 5. Conclusions

In this work, a comprehensive spectroscopic study of Tb^3+^-doped CaF_2_ single crystals with different TbF_3_ concentrations has been performed, combining optical absorption, Judd–Ofelt analysis, photoluminescence spectroscopy, fluorescence dynamics, colorimetric evaluation, and emission cross-section and gain analysis. The results provide a consistent and detailed picture of the radiative behavior of Tb^3+^ ions in a fluorite-type host in the visible spectral range and clarify the role of concentration-dependent effects on their optical performance. The optical absorption spectra reveal well-defined 4*f*–4*f* transitions of Tb^3+^ ions, indicating that the average local crystal-field environment remains largely preserved. The Judd–Ofelt analysis yields intensity parameters Ω_2_, Ω_4_, and Ω_6_ typical of fluoride hosts, confirming the predominantly ionic nature and relatively high symmetry of the CaF_2_ lattice. The good agreement between experimental and calculated line strengths validates the reliability of the Judd–Ofelt formalism for describing the absorption and radiative properties of Tb^3+^ ions in CaF_2_. The emission spectra are dominated by the ^5^D_4_ → ^7^F_J_ transitions (J = 3, 4, 5), with intense green emission arising from the ^5^D_4_ → ^7^F_5_ transition. The absence of ^5^D_3_ emission, even under direct excitation into the ^5^D_3_ level, is attributed to efficient cross-relaxation processes that transmit excitation energy toward the ^5^D_4_ level. This mechanism, together with the low phonon energy of the fluoride host, enables efficient radiative decay and stable green luminescence over a broad concentration range. Fluorescence lifetime measurements show millisecond-scale decay times characteristic of Tb^3+^ in low-phonon-energy hosts, which changes slightly with Tb^3+^ concentration. The experimentally determined quantum efficiencies increase markedly from low to intermediate Tb^3+^ concentrations and tend to saturate at 10 mol% doping level, demonstrating the robustness of CaF_2_ as a host for efficient Tb^3+^ emission. The color chromaticity analysis confirms that all samples emit in the green region with good color stability, governed by the dominance of the ^5^D_4_ → ^7^F_5_ transition. Emission cross-section and gain parameter calculations further quantify the radiative potential of the investigated crystals, especially for the orange band.

The present study demonstrates that CaF_2_:Tb^3+^ single crystals combine favorable intrinsic spectroscopic properties with a high tolerance to dopant concentration, making them attractive candidates for green-emitting photonic applications.

## Figures and Tables

**Table 1:** Mean wavelengths (λ_m_) and squared reduced matrix elements, [U^(t)^]^2^ (t = 2, 4, 6), for selected optical transitions of Tb^3+^ ions originating from the ^7^F_6_ ground state, used in the JO analysis.

Transition, ^7^F_6_→	$\lambda_{m}$ (nm)	[*U*^(2)^]^2^	[*U*^(4)^]^2^	[*U*^(6)^]^2^
^7^F_3_	2249	0	0.2142	0.3804
^7^F_2_	1952	0	0.0479	0.4676
^7^F_1_ + ^7^F_0_	1826	0	0	0.5178
^5^D_4_	486	0.0007	0.0013	0.0011

**Table 2:** Experimental and calculated electric dipole line strengths for selected optical transitions of Tb^3+^ ions in CaF_2_ crystals doped with 1, 5, and 10 mol% TbF_3_. The mean wavelengths, *λ*_m_, Tb^3+^ ion concentrations, and root-mean-square deviations, *δ*, δ obtained from the JO fitting are also reported.

TbF_3_ Concentration (mol%)		1			5			10		
Transition, ^7^F_6_**→**	$\lambda_{m}$ (nm)	${\left(S_{JJ\prime }^{DE}\right)}_{meas}$	${\left(S_{JJ\prime }^{DE}\right)}_{calc}$	δ	${\left(S_{JJ\prime }^{DE}\right)}_{meas}$	${\left(S_{JJ\prime }^{DE}\right)}_{calc}$	δ	${\left(S_{JJ\prime }^{DE}\right)}_{meas}$	${\left(S_{JJ\prime }^{DE}\right)}_{calc}$	δ
		(×10^−20^ cm^2^)								
^7^F_3_	2249	1.19024	1.11444	0.43	2.03022	1.99188	0.22	1.96614	1.93776	0.16
^7^F_2_	1952	0.77713	1.11609		1.37178	1.54323		1.44786	1.57478	
^7^F_1_ + ^7^F_0_	1826	1.42381	0.84708		1.61265	1.07274		1.63884	1.11539	
^5^D_4_	486	0.00936	0.00936		0.0103	0.01030		0.01538	0.01538	

**Table 3:** JO intensity parameters Ω_2_, Ω_4_, and Ω_6_ (in units of 10^−20^ cm^2^) and the asymmetry ratio χ = Ω_4_/Ω_6_ for CaF_2_:x mol% TbF_3_ crystals at different Tb^3+^ concentrations (x = 1, 5, and 10). Literature data for related fluoride hosts are included for comparison. The fitting errors obtained from the JO analysis are also presented.

CaF_2_:x mol% TbF_3_	Ω_2_ (10^−20^ cm^2^)	Ω_4_ (10^−20^ cm^2^)	Ω_6_ (10^−20^ cm^2^)	χ = Ω_4_/Ω_6_	Ref.
x = 1	7.626 ± 1.927	1.178 ± 0.543	2.266 ± 0.378	0.52	This paper
x = 5	2.397 ± 0.975	4.202 ± 0.275	2.869 ± 0.191	1.46	
x = 10	10.319 ± 0.721	3.747 ± 0.203	2.983 ± 0.141	1.26	
CaF_2_:2 mol% TbF_3_ + 3.2 mol% YbF_3_	4.5708	0.418	3.4958	0.12	[41]
CaF_2_:5% TbF_3_	1.71	2.65	2.25	1.18	[17]
LiYF_4_	28.30	1.65	2.15	0.77	[40]
KY_3_F_10_	3.922	0.299	3.022	0.10	[20]
KYbW	1.909	2.414	4.911	0.49	[47]

**Table 4:** Radiative transition probabilities, *A*_*JJ*′_, branching ratios, *β*_*JJ*′_, and radiative lifetimes, *τ*_rad_, for the ^5^D_4_ → ^7^F_J_ transitions of Tb^3+^ ions in CaF_2_ crystals doped with 1, 5, and 10 mol% TbF_3_, calculated using the JO formalism. Literature values are included for comparison.

TbF_3_ (mol%)		1				5				10			
^5^D_4_→	$\lambda_{m}$ (nm)	*A_JJ’_* (s^−1^)	β*_JJ’_*	τ_rad._ (ms)	σ_em_ (×10^−20^ cm^2^)	*A_JJ’_* (s^−1^)	β*_JJ’_*	τ_rad._ (ms)	σ_em_ (×10^−20^ cm^2^)	*A_JJ’_* (s^−1^)	β*_JJ’_*	τ_rad._ (ms)	σ_em_ (×10^−20^ cm^2^)
^7^F_0_	678	1.73	0.0053	3.05 2.082 [47]		6.17	0.0327	5.29 6.77 [36]		5.50	0.0123	2.23 1.805 [45]	
^7^F_1_	668	2.05	0.0063			7.30	0.0386			6.51	0.0145		
^7^F_2_	650	9.88	0.0302			4.42	0.0233			14.03	0.0313		
^7^F_3_	622	98.93	0.3021		0.48	39.02	0.2065		0.25	134.05	0.2993		0.57
^7^F_4_	585	8.39	0.0256		0.23	13.66	0.0723		0.12	15.40	0.0344		0.28
^7^F_5_	542	189.46	0.5785		0.17	99.63	0.5271		0.10	244.72	0.5459		0.24
^7^F_6_	486	17.09	0.0521			18.79	0.0994			28.06	0.0626		

**Table 5:** Experimentally measured luminescence decay times, *τ*_meas_, of the ^5^D_4_ excited state of Tb^3+^ ions in CaF_2_ crystals doped with 1, 5, and 10 mol% TbF_3_, determined for the main ^5^D_4_ → ^7^F_J_ emission transitions. Literature values reported for Tb^3+^-doped related fluoride systems are included for comparison. Quantum efficiency, η (%).

TbF_3_ Concentration (mol%)		1	5	10	Ref.							1	5	10
					[35]	[33]	[34]	[45]	[36]	[43]	[20]			
Transition, ^5^D_4_→	$\lambda_{m}$ (nm)	τ_meas._ (ms)										Quantum Efficiency, η (%)		
^7^F_0_	678	5.5	5.5	4.9								31.4	71.2	73.9
^7^F_1_	668	5.0	5.6	6.5										
^7^F_2_	650	4.4	5.6	5.8										
^7^F_3_	622	6.2	6.4	6.5										
^7^F_4_	585	6.1	6.4	6.7										
^7^F_5_	542	6.1	6.4	6.7	2.39	7.4	8.1	1.43	6.06	4.2	6.81			

**Table 6:** The CIE color coordinates.

CaF_2_:*x* mol% TbF_3_	λ_exc._ (nm)	x-Coordinate	y-Coordinate	λ_exc._ (nm)	x-Coordinate	y-Coordinate
*x* = 1	378	0.34270	0.54708	486	0.39573	0.59402
*x* = 5		0.34214	0.57734		0.37756	0.61243
*x* = 10		0.35118	0.58089		0.35118	0.58089

## Data Availability

The original contributions presented in this study are included in the article. Further inquiries can be directed to the corresponding author.
